# Activity of the masticatory muscles and occlusal contacts in young adults with and without orthodontic treatment.

**DOI:** 10.1186/s12903-015-0099-2

**Published:** 2015-10-06

**Authors:** Aneta Wieczorek, Jolanta E. Loster

**Affiliations:** Department of Dental Prosthetics, Institute of Dentistry, Jagiellonian University, ul. Montelupich 4, 31-155 Kraków, Poland

**Keywords:** Electromyography, Young adults, Masticatory muscles, Orthodontically treated, Nonorthodontically treated

## Abstract

**Background:**

Symmetry evaluation of the craniofacial complex generally involves models of mandibular movement and masticatory muscle activity, especially during the development of the craniofacial complex.

The aim of this screening study was to detect differences in the asymmetry and activity indices and in the occlusal contact distribution in groups with and without orthodontic treatment, and between the sexes in the healthy population.

**Material and Method:**

This screening study involved the participation of 149 Caucasian (*F* = 101, *M* = 48) 18-year-old volunteers, of whom 77 had received orthodontic treatment (Group I) and 72 had not (Group II). All participants underwent sEMG recording with an eight-channel electromyograph (Bio EMG III). A T-Scan III device was used to analyze the occlusal contact points.

We measured the voltage of the right and left temporalis anterior (RTA, LTA) and of the right and left masseter muscles (RMM, LMM). On the basis of the Naeije study, we calculated the Asymmetry and Activity indices (AsI, AcI).

**Results:**

No significant differences were found in the asymmetry or activity indices, or in the occlusal contact distribution of young adult subjects with or without orthodontic treatment.

There were two findings in the females. First (*p* = **0.04**), a higher voltage (131.12 μV) was recorded in the right temporalis anterior muscle in female group, compared to 119.65 μV in the male group. Secondly (*p* = **0.002**), the activity index showed a predominance of the temporalis anterior (AcI = 10.52). In the males, the activity index showed a predominance of the masseter muscles (AcI =−1.22).

**Conclusions:**

The null hypothesis was supported, as we found that no significant differences were observed in occlusal contact, asymmetry, or activity indices between healthy young adults with or without orthodontic treatment. However, there do exist significant differences in the activity index between genders.

## Background

Asymmetry is defined as the absence or lack of symmetry or balance, and as a dissimilarity of corresponding parts or organs on opposite sides of the body [1]. Asymmetry is a common finding in the human body. The morphology and function of the paired structures on each side differ [2]. Symmetry evaluation of the craniofacial complex generally involves models of mandibular movement and masticatory muscle activity, especially during the development of the craniofacial complex [3–8]. In the studies of Dahlstrom et al. and Ferrario et al., greater muscular activity was found in the masseter in males and in the temporalis anterior in females during clenching [9, 10]. However the number of participants in the study groups was small and inhomogeneous in age, and it has not been determined whether the differences observed in the muscular activity are related to occlusal trauma. In Ferrario’s study, the subjects were dental students aged 20–27 years. The exclusion criteria was the absence of moderate or severe clinical mandibular disorders, no TMJ sounds, no tenderness to palpation of the TMJ or masticatory muscles, and no painful limitations of mandibular movements [10]. These criteria presumably include patients with some clinical symptoms of mandibular disorders, which could influence the measured voltage of the masticatory muscles. The study of Manfredini [11] showed clearly that subjects possessing some symptoms from axis I of the research diagnostic criteria for temporomandibular disorders (RDC/TMD) have lower masticatory muscle voltages than in asymptomatic subjects. The investigated group has a mean age of 34 ± 9 years. Wearing of the teeth can be seen at this age, and this effect may have some influence on the pattern of the function of the stomatognathic system, and thus on the voltage of the masticatory muscles. It would be interesting to determine whether the findings of Ferarrio also hold true for an 18-year-old homogeneous asymptomatic group (according to the RDC/TMD protocol) and to determine the extent of the influence on muscle activity of orthodontic treatment aimed at obtaining balanced occlusion.

The null hypothesis is that the differences in the distribution of the occlusal contacts, asymmetry index, and muscle activity in subjects who have had orthodontic treatment are no different from those in nonorthodontically treated subjects.

The aim of the study was to detect any differences in the asymmetry and activity indices and occlusal contact distribution in groups with and without orthodontic treatment, and between the sexes in the healthy population.

## Material and Method

This screening study involved the participation of 149 Caucasian (*F* = 101, *M* = 48) 18-year-old volunteers, of whom 77 had received orthodontic treatment (Group I) and 72 had not (Group II). Group I consisted of 57 females and 20 males. On the base of the clinical examination, Group I contained 55 people with Class I malocclusions, 12 with Class II malocclusions, and five with Class III malocclusions. Group II consisted of 44 females and 28 males. Of these, 47 had a Class I malocclusion, 18 has a Class II malocclusion, and seven had a Class III malocclusion. They were invited to participate in a project aimed at evaluating the status of the stomatognathic system in healthy young individuals (no. N N403 589138). All subjects were examined clinically by the same trained dentist and answered the Polish version of the temporomandibular disorder questionnaire RDC/TMD [12, 13]. Additionally, they were asked whether they had received orthodontic treatment. Based on the answers, the subjects were divided into an orthodontically treated group and a nonorthodontically treated group. Additionally, a panoramic radiographic image was made with a Frankfurt horizontal plane, with a cephalostat being used to align the head in that position. The radiographs were made with ProMax radiograph unit (Planmeca, Helsinki, Finland, 2005) and acquired at 74 kVp and 10 mA. All x-rays were carried out by the same technician. The x-rays were analyzed by a specialist experienced in maxillofacial radiology.

All the subjects were selected from two high schools in Kraków and were qualified for the study only if they had no past contact with either of the researchers involved in the investigation or with the instruments under investigation. These inclusion criteria were set to avoid any potential bias resulting from preconceived ideas. The study was initiated once the subjects had signed informed consent forms and the research program had been approved by the Ethical Committee of Jagiellonian University. The research was conducted in accordance with the Declaration of Helsinki ICH Guidelines for Good Clinical Practice.

The exclusion criteria were as follows:

1. transverse malocclusion,
2. any periodontal pathology,
3. carious or damaged dental tissues,
4. fixed restorations,
5. bruxism (diagnosed on the basis of parafunctional facets or history of parafunctional tooth clenching or grinding),
6. neuropathic conditions,
7. systemic or localized maxillofacial disease,
8. any pathology or asymmetry in craniofacial structures,
9. Botox therapy,
10. psychological disorders, or
11. pregnancy.

The inclusion criteria were:

1. a full dental arch (volunteers without extraction during orthodontic treatment),
2. no symptoms of TMD based on an RDC/TMD examination,
3. remaining dental midline based on RDC/TMD examination, and
4. symmetry of facial structures based on a panoramic x-ray.

All the study participants underwent a surface electromyography (sEMG) recording with a commercially available device, an 8-channel BioEMG III BioPAK Measurement System Electromyograph (BioResearch, Inc., Milwaukee, WI, USA). Surface EMG signals were obtained from four of the 8 channels, which can record electrical (biopotential) activity from eight muscles simultaneously. Microvolt signals were amplified with minimal noise to 5000 times their original levels. Noise was reduced by 40 dB (equivalent to a 99 % reduction in noise amplitude on a linear scale) using the NoiseBuster digital filtering in the BioPAK program, which automatically removes 99 % of any remaining 50/60 Hz noise (and harmonics) still present in the recorded data at analysis [14, 15]. All recording was carried out in line with the Standards for Reporting EMG Data [16]. A T-Scan III evolution 7.01 device (Tekscan Inc., South Boston, MA, USA) was used to analyze occlusal contact points [17].

All examinations were performed before school, between 8 am and 10 am. Prior to the examination, volunteers were seated for 5 min in a quiet environment listening to relaxing music. The subjects were informed of the aim of the test so that they could offer maximum cooperation. They were instructed to sit upright on a chair with the head unsupported. After the skin was cleaned with 95 % alcohol and rubbed with abrasive paper, the recording was performed using bipolar surface electrodes (BioFLEX, BioResearch Associates, Inc., Brown Deer, WI, USA). The surface BioFLEX EMG Ag/AgCI electrodes with two conductive–adhesive polyester RG-63X hydrogel contacts (100 mm^2^, 19 mm center-to-center interelectrode spacing) were placed bilaterally on the subject’s skin, overlying the anterior temporalis—that is, vertically along the anterior muscular margin and approximately over the coronal suture. For the masseter, the electrodes were placed parallel to the muscle fibers, with the upper pole of the electrode at the intersection between the tragus–labial commissure and the exocanthion–gonion lines, perpendicular to the skin surface, according to the technique described by Ferrario et al. [18]. A plate ground electrode was secured to the forehead [19] and the correct size of sensor (large or small) for the subject was selected, according to jaw size. The sensor was placed in the subject’s mouth with its support pointer between the upper central incisors. The volunteers were then asked to close their jaws, and the T-scan and EMG recordings were started simultaneously. While the recording was in progress, the real-time status bar of both the T-scan and sEMG were shown on the same computer screen [20]. During the examination, the subject was asked to clench the teeth as hard as possible, three times for 3 s each, with 3 s relaxation between each clench [21]. All the recordings were repeated three times. The sEMG examination involved recording the occlusion with the use of a T-Scan III device. All the sEMG and T-Scan measurements were carried out by the same investigator, who possesses expertise in the use of such devices. This protocol is fully described in the article of Wieczorek et al. [22].

To analyze any correlation between the maximum voltage of the masseter muscle (MM) and of the temporalis anterior (TA) at the point at which occlusal contact reached 100 %, T-scan III software was used. This automatically determined the still image with maximal occlusal contact. The first clench of the second registration was chosen for analysis. We selected the sEMG values for analysis during the moment of maximum occlusal contact. The T-scan III software automatically calculated the distribution of occlusal contact at the point of maximum clench when 100 % of the possible contact were reached. The software used is capable of displaying both the maximum occlusal contact achieved and its distribution to right and left, in relation to the midline. The study protocol assumed a homogeneous analysis of the left side, where values of <50 % mean occlusion on the right side, and values >50 % mean occlusion on the left. The T-Scan III/BioEMG III Integration Software showed the maximum voltage of muscles at that moment, and brought the data up from the still of the T-Scan.

Measurement variability was assessed through repeated sEMG analysis, as described in a previous article of Wieczorek et al. [22, 23]. The accuracy and precision were calculated by means of the protocol used by Ferrario et al.—namely, intraclass correlation coefficient (ICC) analysis [24].

To describe the asymmetry of the masticatory muscles, an asymmetry index was calculated using the following formula, where “RMS” refers to the root mean square.

Asymmetry index (AsI) = (RMS_right_ – RMS_left_)/(RMS_right_ + RMS_left_) × 100

The asymmetry index can vary between +100 and−100, with an AsI of +100 describing only right (R) muscle activity, −100 meaning only left (L) muscle activity, and 0 meaning equal left and right muscle activity.

The activity index was used to indicate the relative contributions of the masseter and temporalis anterior muscles in the clenching effort using the following formula:

Activity index (AcI) = (RMS_masseter_ – RMS_temporal_)/(RMS_masseter_ + RMS_temporal_) × 100.

The activity index also varies between +100 and −100, with a value of +100 showing activity of the masseter muscles (MM) only, −100 showing activity of the temporalis anterior muscles (TA) only, and 0 indicating equal activity of both muscle groups.

Both these indices have been previously described by Naeije et al. [5].

The data analysis involved the following steps

1. Measurement variability.
2. Normality distribution test.
3. Comparison between Group I and Group II subjects.
4. Comparison between female and male subjects.

### Ethical approval

This protocol was approved by the Ethical Committee of Jagiellonian University, Poland (approval number KBET/89B/2009). Informed consent was obtained from each participant at the beginning of the study prior to confirmation of their eligibility for the study. The participants were permitted to withdraw from the study at any time and for any reason without prejudice.

### Statistical analysis

All data were analyzed using SPSS Statistics 17.0 (2008) for Windows. Data normality was tested using the Kolmogorov–Smirnov test (with Lilliefors correction) and the Shapiro–Wilk test. For nonnormal data, the nonparametric Mann–Whitney and ANOVA tests were used. For normal distributions, Student’s *t*-test was used. The Intraclass Correlation Coefficient (ICC) was tested using the *F*-test. The level of statistical significance was set at 5 % (*p* <0.05).

## Results

### Measurement variability

The ICC of measurement variability was calculated to be 0.765.

### Normality distribution test

The normality of the data was analyzed using the Kolmogorov–Smirnov test (with Lilliefors correction) and the Shapiro–Wilk test. Due to the significance of the RTA, LTA, RMM, and LMM, the nonparametric Mann–Whitney test (*p* <0.05) was used. Due to the insignificance of the asymmetry index, activity index, and occlusal contacts, the normal distribution of the results was analyzed using the parametric Student’s *t-*test (*p* <0.05).

### Comparison between Group I (orthodontic treatment) and Group II (nonorthodontic treatment) subjects

The differences between the voltage emitted by the temporalis anterior and masseter muscles in the treated and untreated groups were insignificant (Table 1). We found that the distribution of occlusal contacts shifted to the left side in both groups, but the asymmetry index showed a predominance of the muscles on the right side in both groups. The activity index in both groups was comparable, and showed a dominance of the masseter muscles. None of these data were significant. Next, all the voltage data within the orthodontically treated and untreated subgroups was also checked for difference by gender and occlusal class. As these differences were not statistically significant, we combined the data from all 149 subjects and analyzed it by gender alone.

**Table 1:** Voltage of RTA, LTA, RMM, LMM during maximum clench, Occlusal contacts , asymmetry, and activity index in treated and untreated subjects.

	Group	*n*	Medium	SD	*p* <0.05
RTA	Orthodontically treated	77	127.13 μV	67.18	0.62
	Orthodontically untreated	72	127.74 μV	79.72	
LTA	Orthodontically treated	77	96.22 μV	46.49	0.48
	Orthodontically untreated	72	102.76 μV	55.26	
RMM	Orthodontically treated	77	122.68 μV	63.32	0.63
	Orthodontically untreated	72	127.78 μV	85.18	
LMM	Orthodontically treated	77	119.48 μV	78.90	0.64
	Orthodontically untreated	72	125.22 μV	81.03	
Occlusal contact (left %)*	Orthodontically treated	77	52.12	8.72	0.34
	Orthodontically untreated	72	50.68	9.43	
Asymmetry index*	Orthodontically treated	77	8.10	19.44	0.26
	Orthodontically untreated	72	4.44	19.89	
Activity index*	Orthodontically treated	77	2.52	19.74	0.98
	Orthodontically untreated	72	2.61	23.96	

ANOVA test; *Student’s *t*-test. *P* <0.05

### Comparison between the male and female groups

There were 48 males and 101 females in the entire study group (Table 2). In analyzing the voltage emitted by the muscles during clenching, we observed significant differences between males and females (*p* = 0.04) in the RTA (131.12 μV for the females and 119.65 μV for the males), but not between the sexes in the voltage of the LTA, RMM, or LMM. The only significant value in this part of the analysis (*p* = 0.02) was in the activity index, which showed a predominance of the temporalis anterior muscle in females (AcI = 10.52) and a predominance of the masseter muscle in males (AcI = −1.22). Another interesting finding was that, even though the occlusal contact distribution in both sexes was greater on the left side, the asymmetry index showed a predominance of the right side muscles (masseter and temporalis anterior).

**Table 2:** Voltage of RTA, LTA, RMM, LMM during maximum clench, Occlusal contacts, asymmetry, and activity indices according to gender.

	Gender	*N*	Medium	SD	*p* <0.05
RTA	Males	48	119.65 μV	84.68	**0.040**
	Females	101	131.12 μV	67.30	
LTA	Males	48	94.58 μV	46.42	0.579
	Females	101	101.66 μV	52.89	
RMM	Males	48	138.50 μV	83.04	0.249
	Females	101	118.79 μV	69.57	
LMM	Males	48	135.42 μV	89.54	0.138
	Females	101	116.0 μV	74.26	
Occlusal contact (left %)*	Males	48	51.16	9.39	0.810
	Females	101	51.55	8.95	
Asymmetry index*	Males	48	4.41	18.97	0.414
	Females	101	7.24	20.03	
Activity index*	Males	48	10.52	27.11	**0.002**
	Females	101	−1.22	17.70	

ANOVA test, * t-Student test. *P* <0.05

## Discussion

This study involved a homogeneous group of asymptomatic young adults. The analysis of all the data revealed similar values for the voltage of the temporalis anterior muscles and the masseter muscles in both the treated and untreated subjects—even when the distribution of the occlusal contact in the untreated group was more balanced. In the treated group, occlusal contacts were shifted more to the left side, but the asymmetry index was higher, showing a predominance of the muscles on the right side. Analyzing the activity index revealed a predominance of the masseter muscles in both groups. As both indices are similar in this context, all the voltage data for both the orthodontically treated and the orthodontically untreated groups was checked for difference by gender and occlusal class. As these differences were not statistically significant, we decided to divide the entire group of 149 volunteers according to sex. In this analysis, all voltages were similar except in the RTA, where they were significantly higher for females. Occlusal contact distribution was equal in both sexes and was greater on the left side. Analysis of the asymmetry index in females showed that the absolute values of the right masseter and temporalis anterior muscles were more dominant than in the males (7.238 and 4.408 respectively). A balance of occlusion and of muscular activity was thus not observed in the healthy population. This was also observed by Manfredini et al. in their study [11] comparing the voltage of masticatory muscles in healthy subjects and patients. Those authors showed that the voltages in healthy subjects were higher than in nonhealthy subjects, and that the right-side muscles had higher voltages during clenching. He did not divide the group by sex. The most interesting of our findings was that the temporalis anterior muscles were predominant in females while the masseters were predominant in the males. In our previous study, we compared the data from class I, class II, and class III occlusions, and found no significant differences [23]. We also observed this in both the orthodontically treated and orthodontically untreated subgroups. This nonsignificant difference in Classes was also supported by the study of Miralles et al. [24], in which they found significant differences in the masticatory muscle voltages only in postural activity and during the swallowing of saliva, whereas no difference was observed during maximal voluntary clenching. In comparing only classes I and II, and classes I and III, we also failed to find any significant differences. Only in the comparison of classes II and III did we observe significant differences in the activity index, but the number of class III cases was very small, so this relation needs further study [23]. Analyzing the differences in separated class groups by gender, we found significant differences between classes I and II in the activity index, which showed a predominance of the temporalis anterior and, in class II, for the maximum voltage of RTA [25]. Rodrigues-Bigaton et al. [6] compared the activity and asymmetry indices in women with and without temporomandibular dysfunction. Their asymptomatic group consisted of only 19 women aged 19–40 years (average 24.3 ± 6.1 years). They observed a predominance of the masseter muscle during isometric contraction, but during this study, their volunteers were not clenching “as hard as possible,” as was the case in our protocol. They found that, in the rest position, the temporalis anterior predominated. Ferrario et al. [10], in their study of 20–27-year-old subjects, found a predominance of the temporalis anterior in the females, while in the males there was a predominance of the masseter muscles—results similar to ours. They also found that the indices of most of their subjects were asymmetric, and generally showed a predominance to the right side (as in our study); occlusal contact distribution was similar in both sexes, but the asymmetry indices shifted more to the left side in the female group. The predominance of the masseter muscle in males, and of the temporalis anterior in females, may be related to the results of the study of McNamara et al. [26], who found that the Sella–Nasion Subspinale Angle (SNA) was larger in their male subjects almost by 2.0°, and that the gonial angle might also differ between female and male populations—although this last point required further study. A possible explanation for the predominance of the asymmetry index on the right could be that, in line with the general population, the majority of our study population was right-handed. It would be interesting to know whether chewing and clenching habits are related to handedness. While such a finding would probably be of little general relevance, it might be significant in bruxist patients. We therefore intend to include this question in our future investigations.

To the best of our knowledge, no-one to date has analyzed the distribution of contacts in any group, whether of healthy subjects or patients. We aimed to understand how occlusal contacts are distributed at the moment of maximal clench, which coincides with the release of the maximum tension of the muscles during the elevation of the mandibles avoid any grinding movement of the teeth when biting [27]. One very interesting finding was that the distribution of occlusal contacts was not shifted to the left in any group. In our previous study, we found that, during clenching, the masticatory muscle voltage balances at the moment when 65 % of the occlusal contact is made on the left side. Perhaps the shift in the distribution of occlusal contact is associated with the asymmetry of human body—such as the asymmetry of the mandibles and the asymmetry of the masticatory muscle voltage. In the same study, we found that the differences between the right and left masseter muscles at maximal clench are insignificant; the only significant differences we found were between the right and left temporalis anterior muscles [22], may be responsible for shifting the occlusal contact as a result of the asymmetry of the human body.

## Conclusion

The null hypothesis was supported, as we found no significant differences in occlusal contact, asymmetry, or activity indices between healthy young adults with and without orthodontic treatment. However, there do exist significant differences in activity indices between genders.

## Abbreviations

RDC/TMD: Research diagnostic criteria/temporomandibular disorder
TMD: Temporomandibular disorder
F: Female
M: Male
sEMG: Surface electromyography
EMG: Electromyography
MM: Masseter muscle
RMM: Right masseter muscle
LMM: Left masseter muscle
TA: Temporalis anterior muscles
RTA: Right temporalis anterior
LTA: Left temporalis anterior
RMS: Root mean square
L: Left
R: Right
AsI: Asymmetry index
AcI: Activity index
SNA: Sella-nasion subspinale angle
ICC: Intraclass correlation coefficient
SS: Stomatognathic system
